# Bond Strength to Lithium-Disilicate Ceramic after Different Surface Cleaning Approaches

**DOI:** 10.3290/j.jad.b4874329

**Published:** 2024-01-19

**Authors:** Federico Del Bianco, Claudia Mazzitelli, Tatjana Maravic, Uros Josic, Federica Florenzano, Paolo Baldissara, Lorenzo Breschi, Annalisa Mazzoni

**Affiliations:** a Research Fellow, Department of Biomedical and Neuromotor Sciences, DIBINEM, University of Bologna, Bologna, Italy. Conceptualization, prepared original draft.; b Assistant Professor, Department of Biomedical and Neuromotor Sciences, DIBINEM, University of Bologna, Bologna, Italy. Conceptualization, methodology, prepared original draft.; c Junior Assistant Professor, Department of Biomedical and Neuromotor Sciences, DIBINEM, University of Bologna, Bologna, Italy. Prepared original draft.; d Research Fellow, Department of Biomedical and Neuromotor Sciences, DIBINEM, University of Bologna, Bologna, Italy. Methodology, formal analysis, investigation.; e Research Fellow, Department of Biomedical and Neuromotor Sciences, DIBINEM, University of Bologna, Bologna, Italy. Formal analysis, investigation.; f Associate Professor, Department of Biomedical and Neuromotor Sciences, DIBINEM, University of Bologna, Bologna, Italy. Formal analysis, investigation, reviewed and edited the manuscript.; g Professor, Department of Biomedical and Neuromotor Sciences, DIBINEM, University of Bologna, Bologna, Italy. Methodology, reviewed and edited manuscript, supervision.; h Professor, Department of Biomedical and Neuromotor Sciences, DIBINEM, University of Bologna, Bologna, Italy. Reviewed and edited the manuscript, supervision.

**Keywords:** lithium-disilicate glass-ceramic, surface cleaning, decontamination, hydrofluoric acid, ceramic primer, shear bond strength

## Abstract

**Purpose:**

To evaluate the effect of different lithium-disilicate (LiSi) glass-ceramic surface decontamination procedures on the shear bond strength (SBS) to resin cement.

**Materials and Methods:**

Seventy CAD/CAM LiSi ceramic specimens (IPS e.max CAD, Ivoclar) were cut and sintered. Fifty specimens were treated with 5% hydrofluoric acid (HF) for 20 s, while 20 were left untreated. All 70 specimens were then contaminated with human saliva and try-in silicone paste. The following surface cleaning methods were investigated (n = 10): C: water rinsing (control); PA: 37% H_3_PO_4_ etching for 20 s; E: 70% ethanol applied for 20 s; CP: cleaning paste (Ivoclean, Ivoclar) brushed for 20 s; HFSEP: self-etching ceramic primer (Monobond Etch&Prime, Ivoclar) rubbed for 20 s; HF: 5% HF applied for 20 s or no HF etching prior to contamination; SEP: self-etching ceramic primer rubbed for 20 s and no HF etching prior to contamination. Composite cylinders were created and luted with an adhesive resin cement to the decontaminated surfaces. After storage for 24 h at 37°C, the SBS test was conducted. Two fractured specimens per group were observed under SEM to perform fractographic analysis. The data were statistically analyzed with p set at <0.05.

**Results:**

The type of surface cleaning approach influenced bond strength (p < 0.001). HFSEP, SEP, and HF attained higher SBS (p < 0.001) compared to other groups. None of the approaches were able to completely remove contaminants from the ceramic surfaces. SEM images showed residual traces of contaminants on CP-treated surfaces.

**Conclusions:**

The self-etching ceramic primer enhanced bond strength to contaminated LiSi ceramic surfaces, irrespective of previous treatment with hydrofluoric acid.

Computer-aided design and computer-aided manufacturing (CAD/CAM) represent the last frontiers of dental technology for the fabrication of ceramic restorations. CAD/CAM lithium-disilicate (LiSi) ceramics have revealed more uniform surface characteristics and less susceptibility to discoloration compared to traditionally fabricated ceramics,^9,11^ making them materials of excellence for the manufacturing of reliable restorations with high esthetics.^18^

The quality of the bond between ceramic restorations and tooth substrates relies on the luting procedures. At this stage, selection of the most suitable cements and ceramic surface treatment is vital for the achievement of enhanced bonding performance and clinical success.^9,14,30^ Universal adhesives in combination with resin cements have demonstrated good in-vitro effectiveness and clinical performance when used for bonding to LiSi ceramic restorations.^4,12^ In general, adhesion to ceramic restorations occurs after treatment of the restoration with hydrofluoric acid followed by silanization. The microporosities and surface modifications due to acid etching in addition to the chemical coupling provided by silane contribute to highly retentive patterns.^6^ Traditionally, etching is performed by a dental technician prior to delivery to the dental office, reducing chairside time. Alternatively, this procedure can also be performed by the dentist. Despite the benefits named above, hydrofluoric acid must be handled with care, especially in a clinical environment, since it has corrosive potential and can cause skin burns due to the fluoride ions it releases upon contact with skin.^23,27^ Moreover, the high reactivity of hydrofluoric acid makes silica-based glass-ceramics easily contaminable.^16^

Contamination is impossible to avoid during the try-in of the prosthesis in the patient’s mouth prior to cementation, as saliva, blood, or try-in silicone paste may be deposited on the ceramic surface and hinder subsequent proper interaction with the luting material. The try-in phase is important to confirm the correctness of fit, marginal adaptation, esthetics, and occlusal parameters of the prosthesis. However, when saliva comes into contact with the ceramic surface, it forms a film which changes the surface properties (e.g., lower surface free energy and decreased wettability) and counteracts the adhesive effectiveness of luting materials.^2,8,31^ Because of these critical adhesion issues, the surface must be cleaned immediately after contamination.

The water rinsing alone does not seem sufficiently remove the salivary biofilm from restorations.^1^ To recompose the material’s characteristics and simultaneously provide surface decontamination, several surface cleaning methods have been employed.^14,28^ Re-etching with hydrofluoric acid is a viable method,^10^ although some authors have hypothesized detrimental over-etching effects on the physical characteristics of ceramics.^21,28^ Recently, new possibilities are emerging with the introduction of new products on the market which have alternative compositions that provide surface etching and priming in just one application.^26^

Determining the most appropriate decontamination strategy for a LiSi restoration is clinically critical for the stability of the luting system and enhancement of the micromechanical and chemical adhesion to dental substrates.^24^ Accordingly, the objective of this laboratory study was to evaluate the influence of different decontamination approaches on the shear bond strength of a multi-step resin cement to LiSi restorations. The null hypothesis tested was that no differences would exist between the different cleaning protocols with regard to the bond strength to LiSi restorations.

## Materials and Methods

### Specimen Preparation

**Fig 1:**
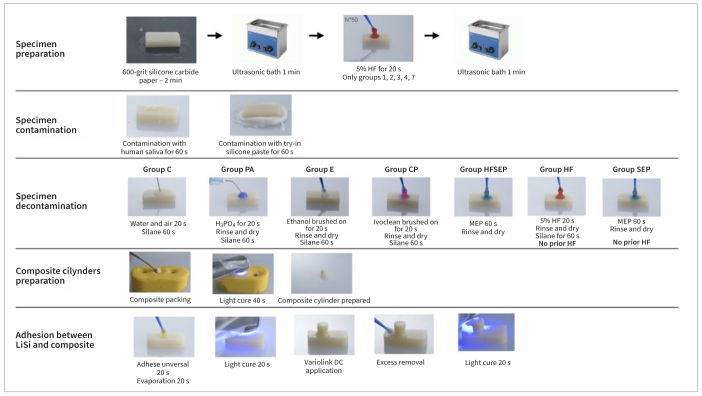
Schematic representation specimen preparation.

The specimen-preparation workflow is illustrated in Fig 1. Seventy ceramic specimens (dimensions 14 x 7 x 3 mm; n = 10, sample size calculated using G*Power 3.1.9.7 for Windows: calculated effect size f = 0.9732105, α = 0.05, power [1-β error probability] = 0.985) were obtained by sectioning lithium-disilicate CAD/CAM blocks (IPS e.max CAD, Ivoclar; Schaan, Liechtenstein) with a slow-speed diamond saw (Micromet, Remet; Bologna, Italy) under water cooling. All specimens were sintered in a laboratory furnace (Programat P500/G2, Ivoclar). Afterwards, the ceramic surface was flattened by wet polishing with #600-grit silicon carbide paper for 120 s. The specimens were ultrasonically cleaned (Transsonic T460/H, Elma Schmidbauer; Singen, Germany) in 50% ethanol for 2 min. Fifty out of seventy ceramic blocks were pre-etched with 5% hydrofluoric acid gel (IPS ceramic gel, Ivoclar) for 20 s, then water rinsed for 1 min and air dried to simulate surface etching as performed by the dental laboratory. These specimens were further ultrasonicated in 50% ethanol for 2 min. The remaining 20 ceramic specimens did not receive any prior hydrofluoric acid etching. The preparation, conditioning, cleaning, and luting procedures were carried out by the same experienced operator (F.D.B.).

All the LiSi specimens (regardless of whether they were previously etched with hydrofluoric acid or not) were contaminated with fresh human saliva obtained from one healthy, male, non-smoking donor who had not taken antibiotics in the previous 3 months and refrained from consuming food and drink the 2 h prior to saliva collection. The saliva was gathered with a cotton pellet and applied to the specimen surface for 1 min.^29^ Then each specimen was pressed with a finger into freshly mixed try-in silicone paste (Fit Checker, GC; Tokyo, Japan) for 2 min. The hydrofluoric acid-etched and then contaminated specimens were randomly allocated to one of the following groups, according to the surface cleaning method (n = 10):

- Group C: only water rinsed for 20 s, followed by air drying for 20 s (control);
- Group PA: 37% H_3_PO_4_ was applied for 20 s, then the specimens were water rinsed and air dried;
- Group E: 70% ethanol was brushed on for 20 s, followed by air drying for 20 s;
- Group CP: a cleaning paste (Ivoclean, Ivoclar) was applied for 20 s, then the specimens were thoroughly water rinsed and air dried for 20 s;
- Group HFSEP: a self-etching ceramic primer (Monobond Etch&Prime, Ivoclar) was rubbed on for 20 s, water rinsed and air dried for 20 s.

The remaining 20 specimens (not etched with hydrofluoric acid prior to contamination) were randomly placed in one of the following groups:

- Group HF: hydrofluoric acid gel (IPS ceramic gel, Ivoclar) was applied for 20 s, rinsed off with water and air dried for 20 s;
- Group SEP: a self-etching ceramic primer (Monobond Etch&Prime, Ivoclar) was applied as in group HFSEP.

A silane coupling agent (Monobond Plus, Ivoclar) was applied for 60 s on the ceramic surfaces and gently air dried for 10 s (groups 1-5), with the exception of groups HFSEP and SEP, in which the self-etching ceramic primer was used.

Two 2-mm layers of a nano-hybrid resin composite (Empress direct; Ivoclar) were compacted into cylindrical silicone molds in order to obtain composite samples (inner dimensions: 4 mm diameter and 4 mm height). Each layer was light cured for 40 s (Bluephase G2; Ivoclar) and, after removal of the composite cylinder from the mold, additional polymerization was performed from all sides for 40 s. The composite cylinders were wet-polished with #600-grit silicon carbide paper for 120 s and then ultrasonically cleaned in 50% ethanol for 2 min.

Further, one composite cylinder was cemented on each ceramic specimen with dual-cure adhesive resin cement system (Adhese Universal and Variolink Esthetic DC; Ivoclar). The universal adhesive was applied on both ceramic and composite surfaces for 20 s and then air dried for 10 s following the manufacturer’s instructions. Afterwards, the resin cement was applied on the composite cylinder and the cylinder was placed on the adhesive-impregnated ceramic surface using finger pressure.^9^ The resin cement excesses were removed with a microbrush, and the surfaces were light cured for 40 s. After polymerization, the specimens were stored in deionized water at 37°C for 24 h before being embedded in self-curing polymethylmethacrylate (PMMA) resin (Technovit 4071, Kulzer; Hanau, Germany), carefully avoiding any resin interference with the bonded interface. After complete setting of the resin, each bonded specimen was submitted to the shear bond strength test (SBS). The ceramic blocks were inserted into a customized specimen support and the SBS test was performed with a blade using a universal testing machine (Instron 4301; Norwood, MA, USA) at a crosshead speed of 0.5 mm/min. Shear forces were applied at the ceramic/composite interface until debonding occurred.

After testing, the debonded specimens were observed under a stereomicroscope at 50X to assess the failure pattern, as follows: adhesive between ceramic and resin cement (A), cohesive within resin cement or composite (C), or mixed when A and C occurred simultaneously (M).

Two specimens per group were randomly selected, sputter-coated with gold-palladium, and observed in a scanning electron microscope (SEM, Jeol; Tokyo, Japan) at different magnifications to evaluate ceramic surface morphologies after SBS testing.

After failing the normality validation (Shapiro-Wilk test), the data were statistically analyzed (SigmaPlot, Systat Software; Chicago, IL, USA) with the Kruskal-Wallis test followed by pairwise multiple comparisons (Dunn’s test) (p < 0.05) by a researcher who was not aware of the group names.

## Results

Table 1 shows the mean shear bond strengths and standard deviations with statistically significant differences and mode of failure of the tested groups.

**Table 1:** Mean shear bond strengths (standard deviations) with the different ceramic surface-cleaning approaches

Cleaning methods	Mean (SD)
C	145.0 (43.0)^c^
PA	130.4 (22.0)^c^
E	128.6 (15.1)^c^
CP	179.9 (44.7)^bc^
HFSEP	195.9 (27.0)^a^
HF	211.0 (39.2)^ab^
SEP	212.3 (20.7)^a^

C: water rinsing; PA: 37% phosphoric acid etching; E: 70% ethanol; CP: 20s cleaning paste; HFSEP: self-etching ceramic primer after 5% hydrofluoric acid etching; HF: 5% hydrofluoric acid etching only after contamination; SEP: self-etching ceramic primer without prior hydrofluoric acid etching. Different lower case letters indicate statistically significant differences between the groups (p < 0.05).

Statistical analysis revealed that the type of ceramic surface cleaning influenced the shear bond strength (p < 0.001). Significantly higher bond strengths were obtained when ceramic primer was used as a cleaning solution (groups SEP and HFSEP), regardless of the previous etching of the ceramic surface with hydrofluoric acid, compared to the CP, E, PA and C groups (p < 0.05). The SEP groups did not, however, differ significantly from group HF, where etching with hydrofluoric acid was performed only after contamination (p > 0.05). HF group did also not differ significantly from the CP group (p > 0.05), but yielded higher bond strengths than were observed in the E, PA, and C groups. The lowest bond strengths were registered when water rinsing, ethanol, and phosphoric acid etching were used as cleaning procedures, with no significant differences between them (p > 0.05).

All groups similarly demonstrated a majority of adhesive fractures at the resin cement/ceramic surface interface. However, mixed failures were observed for the groups SEP, HFSEP, and HF. No cohesive failures were found.

**Fig 2:**
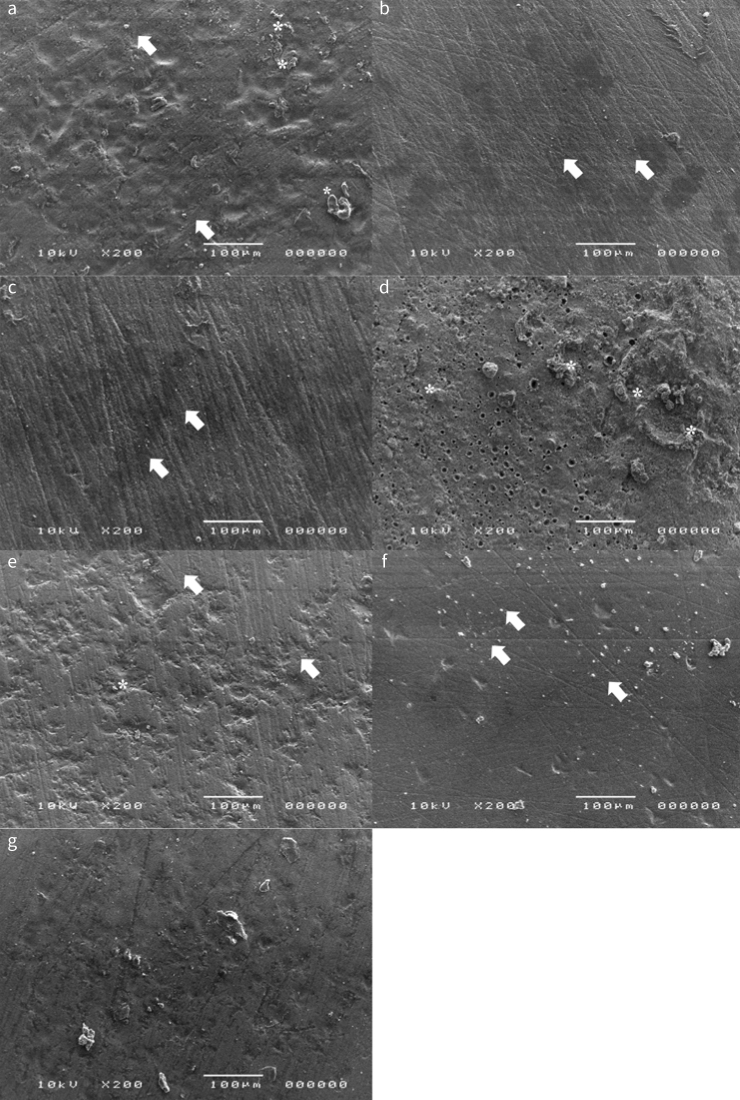
Representative SEM images of LiSi ceramic surfaces after cleaning with the tested methods. a) water rinsing; b) 37% phosphoric acid etching; c) 70% ethanol; d) cleaning paste; e) 5% hydrofluoric acid etching (HF); f) self-etching ceramic primer after HF (HFSEP); g) self-etching ceramic primer (SEP). White arrows indicate the presence of organic contaminants. Traces of silicone remnants were also observed (asterisks).

Figure 2 shows representative SEM images of LiSi ceramic surfaces after decontamination procedures performed in the tested groups. None of the cleaning methods tested resulted in a surface completely free of contaminants. In general, no signs of defects or cracks were observed in the examined LiSi ceramic surfaces. The specimens of the water-rinsed group (C) were smooth and covered by debris (Fig 2a). Resin cement remnants still attached to the ceramic surface were observed in groups SEP, HFSEP, and HF (Figs 2e–2g). Among the groups HFSEP and SEP, a higher degreeof contamination was observed when the primer was applied on an unetched ceramic surface (group SEP). A preponderance of residues of the contaminants covering the entire adhesive surface was observed in group CP (Fig 2d).

## Discussion

The present study investigated the important clinical question of the most suitable cleaning method of LiSi glass-ceramic restorations after try-in procedures and before the final cementation, which could influence the bond strength and longevity of the tooth-restoration complex. According to the results of the present study, the null hypothesis must be rejected since the type of surface cleaning approach influenced the shear bond strength of the resin cement to CAD/CAM LiSi ceramics.

The adsorption of salivary proteins to the surfaces of restorative materials that occurs during the try-in procedures creates an organic coating, which is not readily removed by water rinsing.^28^ Several decontamination methods have been proposed over time, the most common of which consist in scrubbing the intaglio LiSi-ceramic surface with water, ethanol, phosphoric acid, or hydrofluoric acid.^2,14,15^ Hence, we could distinguish between decontamination procedures that comprise only cleaning of the surface, and those which involve both cleaning and etching of the surface, hence inducing topographic changes to the intaglio surface of the restoration. Previous studies found that water rinsing and ethanol were ineffective in removing fluid residues from glass-ceramic surfaces,^2,3^ and incapable of restoring bond strength to LiSi ceramic after saliva contamination.^29^ These results are in accordance with the findings of our study. Hence, alternative extra-oral solutions for decontamination of ceramic, zirconia, or metal restorations have recently been introduced. The cleaning paste investigated in the present study (Ivoclean) is a suspension of zirconium dioxide particles in an alkaline solution. Zirconium dioxide particles have a strong affinity towards phosphates and can therefore absorb salivary phosphate contaminants, leaving behind a clean restoration surface. This alkaline solution has previously obtained good bond strength results when used as zirconia or glass-ceramic surface treatment after contamination with saliva.^2,7,29^ However, in the present study, no differences in bond strength were observed between the cleaning paste and water rinsing, ethanol, or phosphoric acid etching (Table 1). The presence of silicone residues from the try-in silicone paste possibly affected the adhesion to the ceramics, and the solution did not seem able to adequately remove it from the surface (Fig 2). The manufacturers of the cleaning paste recommend not using this solution as a cleaning material in the presence of silicone pastes, in order to avoid interfering with the adhesion mechanisms. In order to standardize the contamination protocol for all the groups, as well as to simulate the daily clinical setting – in which a cleaning paste is often used after the fitting check with a try-in silicone paste – we decided to include this product in our experiments, although we did not use it in complete accord with the manufacturer’s instructions. We hence confirmed the recommendations of the manufacturer and reaffirmed the importance of attentive reading and following the instructions for use.

Etching agents could be more effective in the decontamination of LiSi ceramic surfaces after try-in procedures and offer additional benefits in terms of bonding efficacy. Acid etching of LiSi ceramics changes the topography of the restoration surface, which should increase not only the micromechanical retention of resin cements, but also the chemical bonding by the exposure of silica oxides. The resultant etching pattern depends on the etching agent used. Hydrofluoric acid, a more aggressive compound, creates a more pronounced, porous etching pattern than do phosphoric acid and the self-etching ceramic primer.^17^ Although phosphoric acid etching has been previously described as an effective method to decontaminate ceramic surfaces through acidic dissolution of organic debris,^28,29^ the present study found no differences between cleaning with phosphoric acid, water rinsing, and ethanol, after which specimens were covered with particles that could originate from organic and inorganic components in the contaminants used in the study. Contrary to previous studies, in the present protocol, ceramic surfaces were contaminated with both saliva and try-in silicone paste, which more realistically simulates the clinical try-in procedures. It is highly likely that acidic dissolution of the organic contaminants followed by the removal of silicone debris from the subsequent water-rinse left no opportunity for the acidic gel to create the microroughness necessary to ensure adequate retention with the adhesive. In fact, SEM images showed a rough ceramic surface with grinding lines present, and sparsely distributed, probably bacterial debris.

Another interesting product recently introduced to the market is claimed to simultaneously etch, clean, and prime the surface of LiSi glass-ceramic restorations, providing a milder etching pattern on the surface of the etchable ceramics:^19^ the self-etching ceramic primer is a mixture of ammonium polyfluoride, which etches the ceramic surface, and trimethoxysilylpropyl methacrylate, which is responsible for silanization and presents a milder etching pattern compared to hydrofluoric acid. Variable results in terms of bond strength have been found when compared to the gold-standard pretreatment of lithium-disilicate ceramic restorations, i.e., hydrofluoric acid accompanied by silane application. Namely, several studies demonstrated comparable bonding properties of the ceramic-primer–treated ceramics vs the gold standard treatment,^5,20,25,26^ while other research groups reported the superiority of hydrofluoric acid etching followed by a silane solution.^13,22^ However, in these studies, possible contamination with saliva or silicone pastes was not taken into consideration. To the best of the authors’ knowledge, ours is the first published study in which a ceramic primer has been used as a cleaning approach for LiSi surfaces after both saliva and silicone-paste contamination.

The results of the present investigation showed significantly higher (p < 0.05) adhesion values after decontamination with SEP, both with and without prior HF etching in both SEP and HFSEP groups (Table 1), compared to ethanol, phosphoric acid, or water cleaning. The possibility of using a single product that can both decontaminate and condition surfaces has obvious clinical advantages. The manufacturer claims self-limiting etching capability as a possibility to avoid structural damage of restorative materials. There were no statistically significant differences when a ceramic primer was used, with or without previous acid etching (Table 1), likely confirming the self-limiting effect. However, a higher percentage of organic residues was observed when the simplified primer was used without previous HF acid etching, suggesting that the combined action of the two materials may have greater decontamination effectiveness than the single product alone. Future studies will focus on evaluating the long-term effects of ceramic primer on material bonding and chemistry.

Because contamination of the intaglio surface of an indirect restoration is an absolutely unavoidable event during the try-in procedure of the restoration prior to cementation, and the restoration’s surface must be effectively decontaminated to ensure adequate retention of the resin cement, these findings have important clinical implications. However, the present study also has certain limitations. The current results were obtained only at baseline, but it would be important to evaluate the bond strength of contaminated LiSi specimens cleaned using different protocols also after artificial aging. Furthermore, it would be beneficial to perform an EDX analysis to determine with certainty the composition of the debris found on the bonding interface of contaminated ceramic specimens. The SEM observations on the amount and composition of debris on the specimen surfaces should be cautiously interpreted, since only two samples per group were observed. Lastly, the shear bond strength test, instead of a microshear bond strength test, was performed, which can be considered a limitation of the study.

## Conclusions

The use of a self-etching ceramic primer, with or without pre-treatment with hydrofluoric acid, as well as etching with hydrofluoric acid alone, resulted in improved bond strength to LiSi ceramics after saliva and try-in paste contamination.

## Clinical relevance:

The application of a self-etching ceramic primer could be a viable procedure to effectively clean and prepare lithium-disilicate glass-ceramic surfaces before cementation.
